# From Around the World to Johns Hopkins: International Psychiatry Students’ Learning Through ‘The Perspectives of Psychiatry’

**DOI:** 10.1192/j.eurpsy.2025.2384

**Published:** 2025-08-26

**Authors:** J. Thorman, M. S. Chisolm, K. Elzamzamy, A. J. Huang, P. Zuñiga, D. Roy

**Affiliations:** 1 Scania University Hospital, Malmo, Sweden; 2 Johns Hopkins University School of Medicine, Baltimore, United States; 3 The University of Adelaide, Adelaide, Australia; 4 Catholic University of Santiago de Guayaquil, Guayaquil, Ecuador

## Abstract

**Introduction:**

Whilst the DSM has streamlined research and standardized diagnoses, it faces criticism for falling short in addressing the intricate nature of psychiatric disorders. By relying on checklist-based diagnoses, it overlooks critical dimensions such as personality traits, cognitive functions, the motives driving maladaptive behaviors, and the unique narratives of individuals. In contrast, the Perspectives of Psychiatry (PoP) presents a comprehensive framework that enhances the understanding and treatment of mental health conditions. It integrates diverse origins of psychiatric disorders while emphasizing a thorough evaluation of a patient’s life story. The global reach of PoP, with textbooks translated into multiple languages, has led to the creation of an international fellowship program for clinicians and students worldwide.

**Objectives:**

The speakers will explore the PoP framework and share how this system is 1) implemented at Johns Hopkins (Baltimore, MD) in clinical practice and teaching, and 2) taught to international students with the newly established Perspectives of Psychiatry International Learners Program (PoPPIL).

**Methods:**

Faculty from Johns Hopkins will outline how the PoP has been applied at their institution for the past 40 years, while international students from Sweden, Australia, and Ecuador will share their experiences from the PoPPIL.

**Results:**

The PoP provides a structured and integrated framework for clinical practice and teaching. It explicates the rationale behind various treatment methods — medications, psychosocial interventions, motivational strategies, and consolation — whilst enabling patients to contribute more discerningly in their own recovery. Patients undergo a comprehensive and systematic evaluation, before this information is analyzed from each of the 4 perspectives (figure 1). This encourages clinicians to consider the patient holistically, rather than as a collection of symptoms, leading to the creation of management plans that not only addresses their psychiatric symptoms but also their social needs and personal goals. These plans can then be used to work collaboratively and effectively with other providers such as social services, general practitioners and psychologists. The framework is now part of the core curriculum for all psychiatry residents at Johns Hopkins and since 2024, international students can partake in an immersive learning experience which includes clinical observation and teaching. The PoPPIL has also facilitated grassroot global communication among medical students, junior doctors and leading experts.

**Image 1:**

**Figure:**
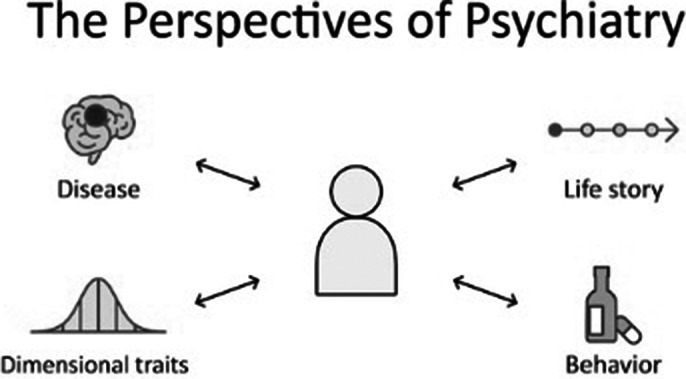

**Conclusions:**

Moving beyond diagnostic checklists to a framework grounded in intelligible concepts can propel advancements in psychiatry. The program at Johns Hopkins illustrates how these concepts can be effectively applied in clinical settings and shared with students from around the world.

**Disclosure of Interest:**

None Declared

